# Shedding Light on Gas-Dynamic Effects in Laser Beam Fusion Cutting: The Potential of Background-Oriented Schlieren Imaging (BOS)

**DOI:** 10.3390/s23020729

**Published:** 2023-01-09

**Authors:** Silvana Burger, Karen Schwarzkopf, Florian Klämpfl, Michael Schmidt

**Affiliations:** 1 Institute of Photonic Technologies (LPT), Friedrich-Alexander-Universität Erlangen-Nürnberg, Konrad-Zuse-Straße 3/5, 91052 Erlangen, Germany; 2 Erlangen Graduate School in Advanced Optical Technologies (SAOT), Friedrich-Alexander-Universität Erlangen-Nürnberg, Paul-Gordan-Straße 6, 91052 Erlangen, Germany

**Keywords:** laser beam fusion cutting, laser material processing, process observation, high-speed imaging, Schlieren imaging, background-oriented schlieren (BOS), gas dynamics, gas jets, shock diamonds

## Abstract

In laser beam fusion cutting of metals, the interaction of the gas jet with the melt determines the dynamics of the melt extrusion and the quality of the resulting cutting kerf. The gas-dynamic phenomena occurring during laser beam cutting are not fully known, especially regarding temporal fluctuations in the gas jet. The observation of gas and melt dynamics is difficult because the gas flow is not directly visible in video recordings and access to the process zone for observation is limited. In this study, the problem of imaging the gas jet from the cutting nozzle is addressed in a novel way by utilizing the striation pattern formed at the cutting kerf as a background pattern for background-oriented Schlieren imaging (BOS). In this first feasibility study, jets of different gas nozzles were observed in front of a solidified cutting kerf, which served as a background pattern for imaging. The results show that imaging of the characteristic shock diamonds of cutting nozzles is possible. Furthermore, the resulting shock fronts from an interaction of the gas jet with a model of a cutting front can be observed. The possibility of high-speed BOS with the proposed method is shown, which could be suitable to extend the knowledge of gas-dynamic phenomena in laser beam fusion cutting.

## 1. Introduction

**Figure 1:**
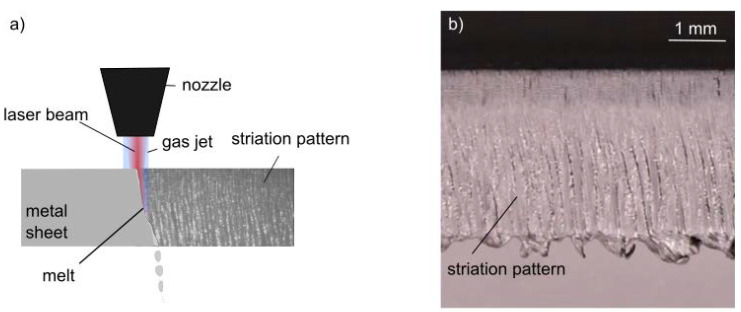
(**a**) Basic principle of laser beam fusion cutting of metals: the material is molten by the laser beam and accelerated by the gas jet. (**b**) Resulting cutting kerf with characteristic striation pattern.

Laser beam fusion cutting is a common application in the material processing of metals. The basic principle for creating cuts is to heat the material for melting it and to remove the melt using a gas jet (see Figure 1a). Despite its many industrial applications, there are still some dynamic effects occurring in this process that are not fully understood. For example, the exact mechanisms affecting the characteristic striation pattern of the resulting cutting kerf (see Figure 1b) have not been fully investigated [1].The interaction of the gas jet with the melt determines the melt extrusion and the process result. In order to further improve the roughness of cutting kerfs and avoid dross at the bottom of the kerfs, a detailed understanding of the dynamic mechanisms regarding the gas flow can be helpful.

The gas flow from a cutting nozzle does not simply consist of a gas flow with uniform velocity and density but underlies more complex gas-dynamic effects. When the gas leaves the cutting nozzle, it expands to a degree, creating an overexpanded jet, which then compresses again. This process creates a characteristic pattern of pressure differences, manifesting in standing shock waves, with a characteristic diamond pattern—so-called shock diamonds or Mach diamonds. The geometry of these shock diamonds depends on the nozzle geometry and the operation parameters [1]. If the gas jet interacts with surfaces, the dynamics become even more complicated. For example, periodic pressure changes in the gas jet can occur due to the interaction with a plane metal surface [2]. A detailed review of the influence of the gas flow in laser beam cutting is given by Riveiro in [1]. There, the need for further investigation of the gas jet is mentioned, especially with a focus on its dynamic behavior.

Imaging of the gas flow is difficult because of the limited accessibility of the process zone, the high velocities of the gas stream, and the transparent gas flow. Therefore, it is challenging to determine the effects of the gas flow on the melt flow and possible feedback effects of the melt flow on the gas flow. Recently, there have been advances in the process observation of the melt flow, for example, with the help of high-speed imaging [3]. For observing the process zone from a side view, typically so-called ‘trim cuts’, which means cutting along the edge of the metal sheet, are performed. In addition, trim cuts can be used in combination with glass setups, where a glass sheet is used as a replacement edge, allowing imaging of the melt flow [4].

There have also been advances in imaging the melt flow of an undisturbed cutting process utilizing high-brilliance synchrotron radiation sources. These allow for high-speed X-ray imaging of the process, currently with frame rates around 1000 fps (frames per second) [5]. In this way, the melt expulsion can be observed, but the dynamics of the gas jet from the nozzle remain out of reach.

Imaging of gas jets from cutting nozzles was conducted in [6] with a classical Schlieren imaging technique. There are various setups for Schlieren imaging. They are all based on the effect of the distortion of light rays, which occurs when light is sent through a volume of interest, where different refraction indices are present. Schlieren imaging can be used for the observation of gas jets as the different pressures in the gas jet cause slightly different refraction indices. Classical Schlieren setups are based on an optical setup, consisting of mirrors or lenses and requiring space around the specimen, where the Schlieren effect can be observed due to a change in path as the light passes through the medium and the Schlieren image is directly generated at a screen or camera sensor. In this study, background-oriented Schlieren imaging (BOS) was used, which is described in detail in Section 2.1. This allows for a simpler setup without additional mirrors or lenses but relies on algorithmic postprocessing of the images.

In [6], classical Schlieren imaging was conducted for the observation of the undisturbed, free gas jet from a cutting nozzle (ex situ), as well as the interaction of the jet with a simple cutting kerf specimen made of glass. The transparent glass model allowed for Schlieren imaging of the gas jet between two glass sheets. The investigations of the undisturbed jet show the characteristic shock diamonds in the gas stream leaving the nozzle. The geometry and position of the shock diamonds depend on the nozzle geometry and the pressure at which it is operated. In the glass model, the shock diamonds as well as the effect of flow separation in the lower area of the model cutting front can be observed. Due to the limited accessibility of the process zone in laser beam fusion cutting, this can only be observed using the glass model. Such a model was also used in [7], focusing on the simulation of the flow separation behavior in the cutting kerf and applying Schlieren imaging in order to compare the results with the flow separation in the glass setup. An analysis of the boundary layer flow is also given in [8]. Both studies focus on the general pressure or velocity distribution but neglect the highly dynamic behavior that can occur in gas jets. Such behavior due to gas jet impingement was, for example, investigated in [2], however in the more general context of fundamental research, not in the context of laser beam cutting. In [2], acoustic phenomena occurring during the impingement of a gas jet onto a perpendicular plate were investigated with high-speed Schlieren imaging. Highly dynamic phenomena such as upstream traveling acoustic waves can be visualized in this way, contributing to the understanding of the dynamic behavior of gas jets.

The approach described in the present study is a first step in the direction of bringing the observation of gas jets closer to the real conditions in laser beam fusion cutting. In this study, background-oriented Schlieren imaging (BOS) was used for imaging of the gas jet from a cutting nozzle in front of a solidified cutting kerf.

It has to be investigated whether the shock fronts from cutting nozzles can be imaged with this technique and whether this technique is additionally applicable in a high-speed imaging context. The investigations in this study focus on the observation approach and could provide the basis for further studies investigating the gas and melt dynamics in laser beam fusion cutting.

Furthermore, the imaging technique as it is shown here already provides a simple and fast but still detailed way of checking the gas jet of the nozzle. This can be useful for monitoring gas jets and identifying deformed or soiled nozzles. As the system technology is simple, this approach could also be applicable in an industrial environment.

## 2. Materials and Methods

### 2.1. BOS Method

**Figure 2:**
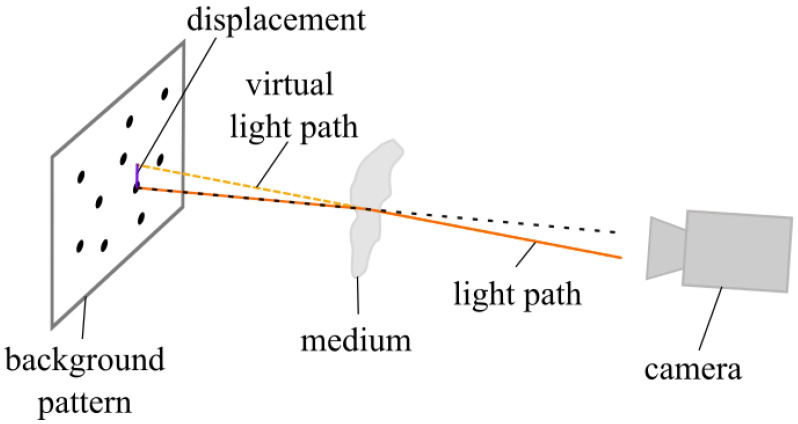
Principle of the background-oriented Schlieren technique (BOS).

The gas jet is imaged by employing the background-oriented Schlieren method (BOS). The BOS method is based on camera observation of a known background pattern, which appears distorted when changes in the diffraction index occur between the camera and background pattern (see Figure 2). In practice, a reference image of the background pattern is recorded, which is than compared with the appearance of the background pattern behind the gas jet. An extensive review of the BOS technique is given in [9]. In the present study of laser beam cutting, the solidified striation pattern from a laser-cut metal sheet is used as a background for BOS.

The changes in refractive index are made visible by algorithmic postprocessing of the images. A simple way of comparing the images of the gas jet together with the background pattern I to the reference image of the background pattern I_ref_ is by pixelwise subtraction of the gray values:

I_BOS_ (*x*, *y*) = *k*∙abs (I (*x*, *y*) − I_ref_ (*x*, *y*)) (1)

where *x* and *y* are the pixel positions in the *x* and *y* directions, abs is the absolute value function, and *k* is a scaling factor for visualization. This way of calculation for the BOS image was used in this study.

Alternatively, more sophisticated image processing algorithms can be used, which are able to recognize as well as quantize the distortion of the background image, for example, optical flow algorithms [10].

**Figure 3:**
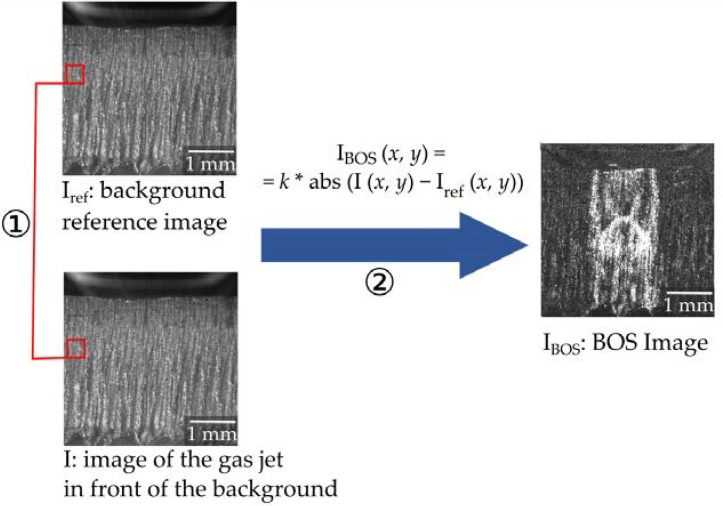
Image processing steps for BOS: (1) preprocessing (optional, depending on the imaging situation): exact alignment and brightness adjustment. (2) Comparison between I and I_ref_ results in the BOS image I_BOS_.

Preprocessing steps can improve the quality of the resulting Schlieren image. As the BOS method is very sensitive to vibrations, in a preprocessing step the exact alignment of each image with the background image can be checked by comparing the grey values in an area where no distortion due to the gas jet is expected. If deviations occur, I and I_ref_ can be exactly aligned algorithmically before the calculation of the BOS image I_BOS_. As for most recordings in this study, vibrations at the setup could be avoided, and no algorithmic alignment was necessary. As a preprocessing step, a slight regulation of brightness was required, as the brightness of the illumination laser changed shortly after the start of operation. Therefore, the average gray value is calculated for an area of the image, where no changes due to the Schlieren effect are expected (see Figure 3 (red)), and the brightness of the whole image I is adjusted to the brightness of the reference image I_ref_.

### 2.2. Observation Setup

In the first step, imaging was conducted with a setup providing good conditions for BOS, such as good accessibility for illumination and observation and measures to avoid vibrations (setup 1). As imaging of shock fronts is possible in setup 1, in the second step imaging was conducted with a laser cutting setup in the laser welding and cutting machine ERLASER UNIVERSAL (setup 2). These experiments provide the basis for future research.

**Figure 4:**
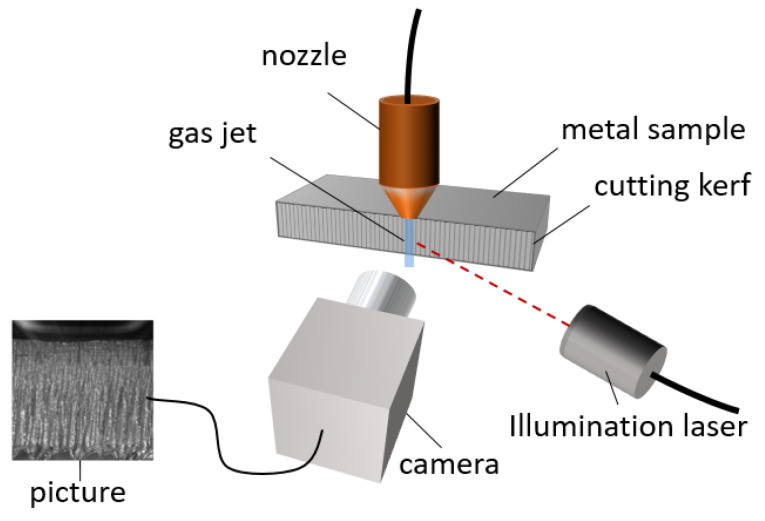
Setup for BOS of the gas jet from the nozzle. The setup consists of the cutting nozzle with its gas jet, which is observed; the cutting kerf of the metal sample acting as a background pattern in BOS; and a camera and illumination system.

The basic imaging setup is shown in Figure 4. It consists of imaging equipment (camera and illumination), a background pattern (the striation pattern of a cutting kerf), and the Schlieren object (the gas jet from a cutting nozzle). In the experiments, a high-speed camera (Phantom v1210, Vision Research Ltd., Wayne, NJ, USA) and an illumination laser (CAVILUX HF, Cavilux) for high-speed imaging were used to investigate the possibilities for observations of highly dynamic effects with this imaging technique. The nozzle is positioned in such a way that the gas flows directly next to the cutting kerf; the exact positioning depends on the respective experiment and is described in the following experiment descriptions.

#### 2.2.1. Setup 1: Observation Test Setup

Setup 1 focuses on the investigation of the feasibility of BOS in the context of laser cutting and provides good conditions for BOS, as shown in Figure 4. The nozzle was used with compressed air at a pressure of 4 bar. The experiments aimed for visualization of typical gas-dynamic effects that play a role in laser cutting, such as the characteristic shock wave structure of the gas jet or the expansion of the vapor plume when metal evaporates. With this setup, the observation of four different phenomena was conducted, the first three being related to the gas jet of the nozzle, the last one to the evaporation in laser cutting:

1. The gas jets of three nozzles with different opening diameters;
2. The process of turning on a nozzle, demonstrating observation with high temporal resolution;
3. The interaction of a gas jet with a model cutting front;
4. The expansion of the vapor plume when locally heating the metal model front with a processing laser.

(1) In the first step, it was tested whether the differences in the gas jets of three nozzles with different opening diameters can be made visible with the proposed technique. Cutting nozzles with opening diameters of 1.0 mm, 1.2 mm, and 1.8 mm were investigated. Therefore, the nozzle opening was set lower than in the normal cutting position so the whole gas jet could be imaged in front of the background. The nozzle was positioned directly in front of the kerf, with the center of the nozzle opening in a lateral distance of about 1 mm in front of the kerf in the background. The valve of the gas tube leading to the nozzle was automatically opened with a magnetic valve after the start of the recording. In that way, the reference background pattern was recorded directly before the operation of the nozzle started, what reduced the possibility of shifts in the camera position or the sample between the recording of I and I_ref_. The influence of vibrations was minimized by the mechanical separation of the camera fixture and the cutting kerf from the mount with the nozzle fixture, which tends to vibrate when the gas valve is opened, and from the computer unit controlling the setup, which can also induce vibrations.

In BOS, the observed displacement of the background due to the Schlieren effect depends on the distance of the gas jet from the background pattern, as directly visible in Figure 2. As a result of the direct proximity of the gas jet and the striation pattern, the displacement due to the Schlieren effect is small, which can make it more difficult to observe small changes in the refractive index.

The images were recorded with frame rates of 37,000 fps at a resolution of 512 px × 512 px, with an exposure time of 1 µs and illumination times of 1 µs or 0.5 µs.

(2) Imaging gas jet fluctuations was tested by observing the inset of the gas flow when the valve of the tube leading to the nozzle was opened. As gas jets show highly dynamic behavior, it is important to enable accurate snapshots if temporal fluctuations are to be visualized. The literature shows that snapshots with illumination times of 2 µs and below are suitable for the observation of dynamic phenomena in gas jets [11]. In order to observe dynamic behavior, the Schlieren effect in this setup must be visible at short illumination times. Therefore, imaging with short illumination times of 1 µs and 0.5 µs was tested.

(3) For research on gas dynamics in laser fusion cutting, the interaction of the gas jet with the process zone is important. As a first step towards the investigation of this interaction, a cutting front model was investigated. The model’s influence on the shock fronts in the gas jet was observed. Therefore, a 1 mm thick, sloped piece of steel, acting as a model cutting front, was clamped to the cutting kerf from the previous experiments. The 1.8 mm diameter nozzle was positioned above the model, like in a laser cutting application. As the gas jet interacts with the metal edge, the shock front pattern alters as depicted in the literature [6]. However, as the gas jet is slowed down by the interaction with the model front, as well as by the friction between the jet and the directly adjacent background striation pattern, it has to be investigated if the pressure differences at the shock fronts are large enough to be visible with the proposed BOS setup.

(4) When metal evaporates in laser cutting, this also leads to differences in refractive index, which can be investigated by Schlieren imaging. In the last experiment with setup 1, a processing laser beam was directed at the model metal front. The laser was operated at a laser power of 1200 W for 120 ms in order to locally melt the model steel front and observe the evaporation with BOS. As a processing laser, the TruDisk6001 was used (wavelength λ=1030 nm) in combination with a 200 µm diameter fiber and a Trumpf BEO D70 processing optics, resulting in a 200 µm spot in the focal plane. An exact alignment of the focal plane and the point of impact at the model front was not performed, as only melting and evaporation at the metal front needed to be achieved. In this first experiment, the model front and the laser beam stayed static (unlike in the cutting application, where either the cutting head or the workpieces are moved). Images were captured at a rate of 73,000 fps with an exposure time and illumination time of 1 µs.

#### 2.2.2. Setup 2: Laser Cutting Setup

**Figure 5:**
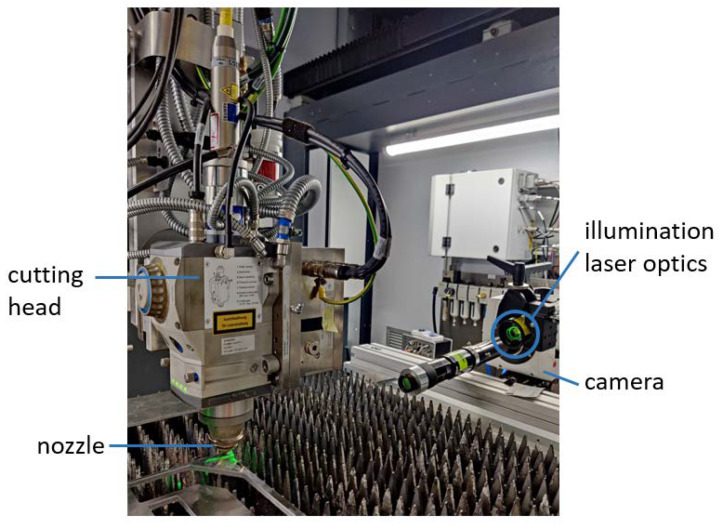
Setup for BOS of the gas jet at the laser cutting machine, consisting of a high-speed camera, an illumination laser, and the cutting head together with a piece of metal from which a slice was cut and removed in order to have a clear view of the striation pattern at the cutting kerf.

As imaging of shock fronts was possible with the experimental observation setup 1, the camera and illumination laser were subsequently integrated into an industrial cutting application setup (setup 2; see Figure 5). For this, the ERLAS laser welding and cutting system was used. The system consisted of a TruDisk 8001 laser with a maximal power of 8 kW and an enclosed housing containing the work area, a cutting head with processing optics, and gas supply. With this system, a 3 mm thick AlMg3 metal sheet was cut at a laser power of 3 kW with nitrogen as a process gas at a pressure of 3 bar. After cutting, the enclosure of the machine was opened and the cutoff piece was removed. This was necessary as the cutoff piece was shielding the cutting kerf from the camera. After this step, the cutting kerf was visible for the camera. Subsequently, the nozzle was moved back to the cutting position over the solidified cutting kerf and the gas jet was turned on again. Nitrogen was used as a process gas, at a pressure of 3 bar. In the cutting machine, the alignment of the illumination laser and camera is more difficult, as is the protection from vibrations. Sparkling reflections of the illumination laser light on the metal surface should be avoided in order to obtain BOS images with good resolution. Images were recorded with frame rates of 37,000 fps.

## 3. Results

With both setups, it was possible to observe shock fronts. The results are shown and discussed in the following.

### 3.1. Shock Diamond Patterns of the Gas Jets from the Nozzle

**Figure 6:**
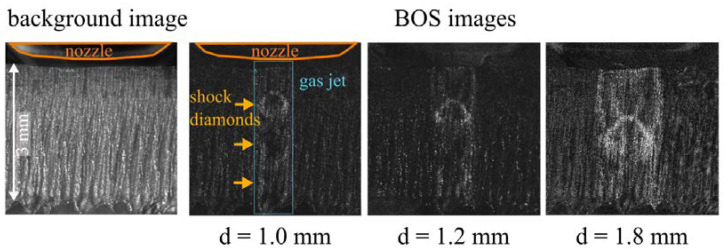
Background image of the striation pattern of a cut metal sheet; BOS images of the gas jets from three cutting nozzles with different opening diameters (d = 1.0 mm, d = 1.2 mm, and d = 1.8 mm). Illumination time: t = 1 µs.

With the first setup, the shock diamonds of the cutting nozzles could be visualized. In the BOS images, the typical pressure difference patterns called shock diamonds, which are caused by the expansion and compression of the gas jet, become visible. Cutting nozzles with opening diameters of 1.0 mm, 1.2 mm, and 1.8 mm were investigated. The differences in their characteristic shock diamond patterns become visible in the evaluated BOS recordings (see Figure 6).

The size of the shock diamonds increases with increasing nozzle opening diameter. The first shock diamond that is directly adjacent to the nozzle is well visible in the BOS images. The shock diamonds further down in the gas jet appear blurry compared with the first one and are only indistinctly visible for the nozzles with 1.0 and 1.2 mm opening diameters. This is expected, as with increasing distance from the nozzle opening, friction effects in the gas jet lead to smaller pressure differences. The position of the first shock diamond can be determined from the images; its distance to the nozzle opening increases with increasing opening diameter. These observations show the potential of the described BOS technique for a simple and fast measurement of the shock diamond position. Due to the existence of the shock fronts in the gas jet, the pressure in the gas jet changes with the distance from the nozzle. This makes the standoff distance between nozzle and metal sheet an important parameter in the cutting application. This was verified by the results in [12], showing that the cut quality is sensitive to the nozzle position.

### 3.2. Fluctuations in the Gas Jet during Starting Operation of the Nozzle

**Figure 7:**
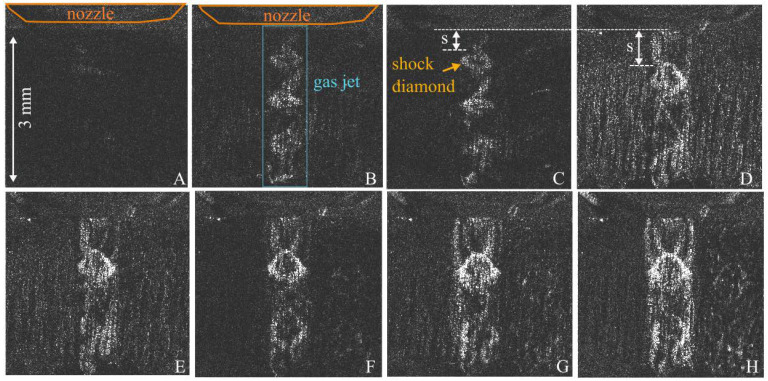
High-speed BOS imaging at the start of operation of the nozzle with a frame rate of 37,000 fps and an illumination time t = 0.5 µs. (**A**) The dark BOS image directly at the start of operation is shown. After the start of operation, fluctuations in the shock fronts are visible for about 30 ms (**B**–**G**), followed by a more steady appearance with the characteristic shock wave structure (**H**).

With the proposed imaging setup, the dynamics during the start of the operation of the nozzle can be visualized, as shown in Figure 7. The start of the operation of the 1.2 mm nozzle was recorded at a frame rate of 370,000 fps and an illumination time of 0.5 µs. Fluctuations in the shock fronts of the gas jet are visible (Figure 7B–G) for around 30 ms, followed by a more steady appearance of the gas jet (Figure 7H). In pictures C–H, it becomes visible how the distance between the nozzle and the first shock diamond slightly increases until it reaches its final static position. These results show the feasibility of the proposed BOS method in combination with high-speed imaging.

### 3.3. Interaction of the Gas Jet with a Model of the Cutting Front

**Figure 8:**
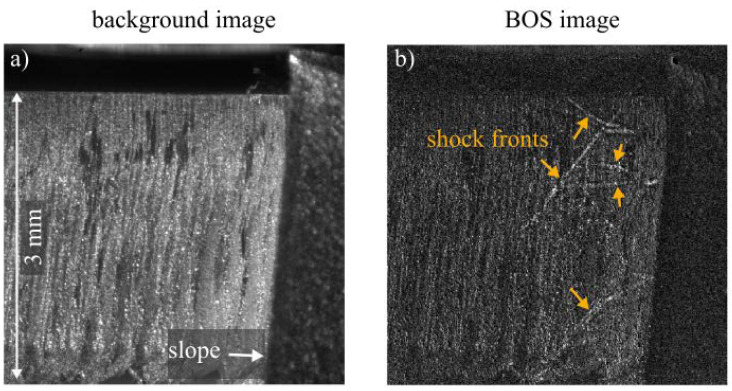
(**a**) background image. (**b**) BOS image of the interaction of the gas jet from the 1.8 mm diameter nozzle with the model of a cutting front, with an illumination time of 0.5 µs. The shock fronts are visible as bright lines.

For research on gas dynamics in laser cutting processes, the interaction of the gas jet with the process zone is important. The initial step to investigate this behavior comprises the investigation of a ‘model front’. Therefore, a metal sheet with a sloped edge and a thickness of 1 mm was clamped in front of the background cutting kerf. For these experiments, the nozzle with a 1.8 mm opening diameter was used. As the shock fronts were not visible in the first experiments with a 0.2 µs illumination time, the illumination time was increased to 0.5 µs. This illumination time is still short enough to generate snapshots for the observation of dynamic effects. After the nozzle is turned on, standing shock fronts, like those depicted in Figure 8, build up. The shock fronts resulting from the interaction with the inclined surface differ visibly from the shock fronts of the free jets in Figure 4 and Figure 5. Similar behavior was visible in the experiments in [6], where a glass model and a conventional Schlieren setup utilizing parabolic mirrors was used. In Figure 8, it can be seen that certain irregularities such as the big dark spots at the cutting kerf are also visible in the BOS image and can locally decrease the quality of the BOS image.

### 3.4. Observation of Evaporation

**Figure 9:**
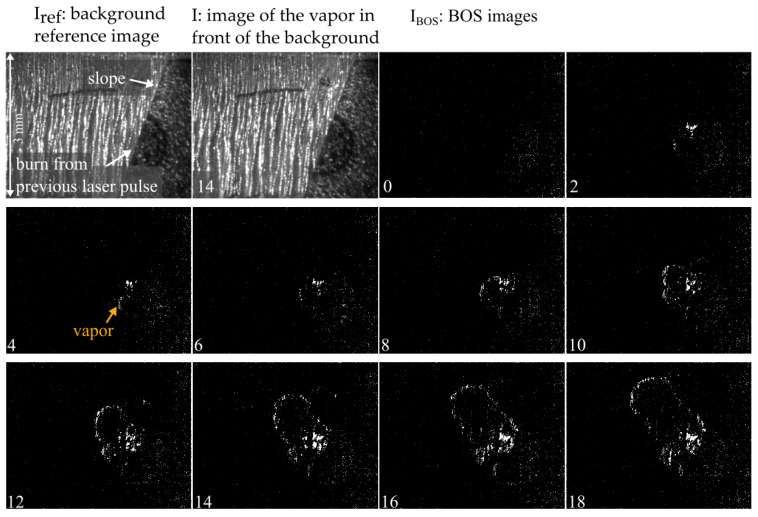
Start of the evaporation when heating the metal front with a processing laser. The expansion of the vapor is not directly visible in the recorded images but becomes visible in the BOS images. The images were captured at a rate of 73,000 fps with an illumination time of 1 µs; every second image is shown here. That means about 27 µs pass between two pictures shown here.

The metal evaporation process at the model front was recorded at a frame rate of 73,000 fps with an exposure and illumination time of 1 µs. A slight flickering is directly visible in the recordings. Apart from that, the evaporation is not visible in the recording and only becomes visible in the evaluated BOS images, as shown in Figure 9. For example, BOS image number 14 is calculated from the first two images in Figure 9: the background reference image I_ref_ and image I_14. In I_14, the expansion of the vapor is not discernible; it only becomes visible in the processed BOS image I_BOS__14.

### 3.5. Observations in the Laser Cutting Machine

**Figure 10:**
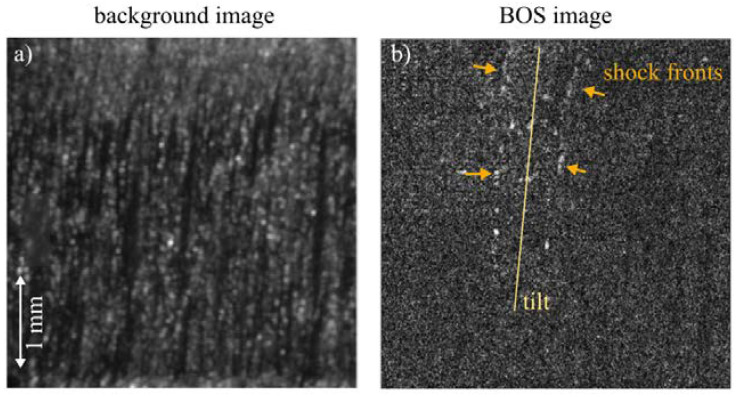
BOS imaging of the gas jet in a conventional laser cutting machine. (**a**) A previously cut kerf is used as a background image. (**b**) The gas jet is imaged afterwards with a frame rate of 37,000 fps and an illumination time of 0.5 µs.

In the BOS images from the laser cutting machine, the gas jet is also visible (see Figure 10). The BOS images from the laser cutting machine show the structure of the shock diamonds less clearly than the BOS images generated with the first observation setup. Possible reasons for this observation are discussed in the following chapter.

## 4. Discussion

With the proposed BOS method, pressure differences in the gas jets of the laser cutting nozzles could be imaged. The results show that the striation pattern of laser-cut metal sheets is a suitable background pattern for BOS. Imaging of the shock fronts was possible despite the proximity of the gas jet to the striation pattern, which weakens the visibility of the Schlieren effect.

### 4.1. Setup 1

The differences in the gas jets of the different nozzles are clearly discernible. In the BOS images, the characteristic shock fronts near the opening of the nozzle show the same typical behavior as observed in [6] with a Z-type Schlieren setup. Comparing the quality of BOS images with conventional Schlieren configurations, conventional methods generate more detailed images. In BOS, the details that can be imaged depend on the background pattern. Future investigations could provide a deeper analysis on how the striation pattern with its vertical structures affects imaging. Furthermore, it can be investigated if imaging can be improved for less reflective materials than AlMg3. Besides aluminum alloys, steel is also used in many cutting applications. Steel is less reflective for the illumination laser light, leading to improved image quality, which is expected to also improve the quality of the BOS images.

In [6], not only the first shock diamond near the nozzle opening is visible but a whole repeating pattern of shock diamonds in the gas jet. This pattern of repeated shock diamonds becomes increasingly blurry with increasing distance from the nozzle opening. However, the BOS setup proposed in this study is relatively simple compared with the setup in [6], and high-speed BOS with the much shorter integration time of 1 µs was performed in this study.

Short integration times of 1 µs or 0.5 µs allow for snapshots of the gas jet and, therefore, imaging of dynamic phenomena.

The results of Section 3.3 in particular, showing the resulting shock waves from the interaction of the gas jet with an inclined plane, have potential for the research on gas dynamics in laser fusion cutting. The shock fronts are visible despite friction losses due to the interaction with the model front and despite the short distance to the background pattern. The setup could be helpful for generating further insights into the gas dynamics in laser cutting, especially if the experiment can be altered in such a way that the interaction with a molten metal front can be investigated. Furthermore, a glass sheet for modeling a cutting kerf should be added, as the gas jet interacts in a different way with a single metal edge and a cutting kerf.

It was demonstrated that this BOS setup can also be used for the observation of evaporation processes, which play an important role in laser cutting. The proposed observation technique could be helpful for the investigation of evaporation effects in classical laser fusion cutting using a gas nozzle [13], or in related processes such as laser remote fusion cutting, which is driven by evaporation phenomena [14]. In [13], the influence of vaporization on the striation pattern is inferred from the experiments, which needs further investigation.

### 4.2. Setup 2

The integration of the observation setup in the laser cutting machine led to BOS images of lower quality than with the previous observation setup. This could be due to the observation situation or due to the gas jet itself. Firstly, the positioning and alignment of the imaging setup in the welding and cutting machine is difficult; a decreased image quality compared with the images from setup 1 is already visible in the background image. The adjustment of the illumination laser is more difficult and, in combination with the highly reflective material AlMg3, leads to bright reflections, which decrease the resolution of the Schlieren images. Regarding the gas jet, the lateral positioning of the gas nozzle is improved in the cutting machine compared with setup 1, as it is set directly at the cutting kerf, in the same position as during processing. As the gas jet is exactly in the cutting position, it might interact with the rough cutting kerf, leading to friction losses in the gas jet and lower pressure differences.

Nevertheless, blurred shock diamonds and the direction of the gas jet are visible. This means that the presented observation technique could be helpful for maintenance checks in industrial cutting applications, such as to control the alignment of the gas jet or the identification of altered gas jets due to deformed or soiled nozzles. This is important for laser cutting, as a tilted nozzle can influence the flow separation behavior at the cutting front, which was shown in [6], where a tilt of the nozzle axis prevented the occurrence of boundary flow separation in the model setup. Despite this fact, in the cutting process a tilted nozzle can decrease the shear forces that act on the melt. Therefore, it might not be desirable in applications.

In our experiments, a high-speed camera together with a powerful illumination unit was used to evaluate its applicability in the observation of highly dynamic processes for research purposes. In an industrial maintenance check, where a high temporal resolution is not needed, less expensive system technology would be sufficient.

## 5. Conclusions

The main conclusions from the investigations are summarized in the following:

(1) A method for the observation of the pressure differences in the gas jets of laser cutting nozzles by using the BOS technique was shown. It was demonstrated that the striation pattern of laser-cut metal sheets is a suitable background pattern for BOS.
(2) Imaging of the shock fronts from different nozzles was possible despite the proximity of the gas jet to the striation pattern in the background, which weakens the visibility of the Schlieren effect.
(3) The potential of the described BOS method for the observation of highly dynamic effects in the gas jets was shown. Therefore, the starting process of the nozzle was imaged, using snapshots of illumination times of 0.5 µs, which are suitable for the investigation of the dynamics in the gas stream.
(4) For cutting applications, the interaction of the gas jet with the cutting front is important. As a first step towards these applications, the interaction with a solid model front was imaged. Although due to friction effects a decrease in the pressure differences at shock fronts was expected, and therefore less visibility of the shock fronts in the BOS image, the imaging of shock fronts was possible.
(5) It was demonstrated that this BOS setup can be used for the observation of evaporation processes, which also play an important role in laser cutting. This observation technique will be helpful for the investigation of evaporation effects in classical laser fusion cutting, or in related processes such as laser remote fusion cutting.
(6) Imaging of a laser cutting machine led to BOS images of lower quality compared with the previous observation setup, but blurred shock diamonds and the direction of the gas jet were visible.

## Data Availability

Not applicable.
